# Supplementary material for: Quantitative Traits of Interest in Apple Breeding and Their Implications for Selection
Source: Plants (Basel). 2023 Feb 16;12(4):903. doi: 10.3390/plants12040903 (PMC9964287; doi:10.3390/plants12040903)
Supplement: Supplementary file 1 [file plants-12-00903-s001.zip › plants-2187391-supplementary.pdf]

plants

MDPI

SUPPLEMENTARY MATERIALS

Quantitative Traits of Interest in Apple Breeding and Their Implications for Selection

Radu E. Sestras [1] and Adriana F. Sestras [2,*]

[1] Department of Horticulture and Landscape, University of Agricultural Sciences and Veterinary Medicine Cluj-Napoca, 3–5 Manastur Street, 400372 Cluj-Napoca, Romania
[2] Department of Forestry, University of Agricultural Sciences and Veterinary Medicine Cluj-Napoca, 3–5 Manastur Street, 400372 Cluj-Napoca, Romania
* Correspondence: adriana.sestras@usamvcluj.ro

Table S1. Origin of apple cultivars obtained at HRS Cluj, used in hybridizations.

No.	Cultivar	Origin
1	Ardelean	Jonathan × Peasgood
2	Ancuța	Jonathan × Mărul de Șugag
3	Aromat de vară	O.P. selection – open pollinated seeds from a hybrid code 1008 (belonging to Golden Pearmain × Jonathan)
4	Feleac	Jonathan O.P. selection (selection in natural hybrids – open pollinated seeds) of Jonathan
5	Roşu de Cluj	Jonathan × Senator

Table S2. Origin of apple selections or cultivars used in testcross hybridizations with the Feleac cultivar as father, resulting in 13 hybrid combinations (offspring families).

No.	Genotype	Origin
1	X-17-19	[218/2 (Golden Pearmain × Peasgood) × Clar alb [1]]
2	X-5-71	[III-VI-5-26 (selection from natural hybrids of Golden Pearmain, open pollinated – O.P.[2], noted from now as Golden Pearmain O.S.[3]) × N.J.40[4]]
3	X-3-8	[III-I-16-16 (Jonathan × Red Astrachan) × N.J.40]
4	III-VI-5-26	Golden Pearmain (O.S.)
5	DSF 3/86	[III-VI-5-26 (Golden Pearmain O.S.) × N.J.46]
6	DSF 3/80	[III-VI-5-26 (Golden Pearmain O.S.) × N.J.46]
7	DSF 7/68	[III-VI-5-26 (Golden Pearmain O.S.) × Camuzat]
8	X-9-69	[III-VI-5-26 (Golden Pearmain O.S.) × 218/2 (Golden Pearmain × Peasgood)]
9	X-9-19	[III-VI-5-26 (Golden Pearmain O.S.) open-pollinated]
10	X-9-70	[III-VI-5-26 (Golden Pearmain O.S.) × 218/2 (Golden Pearmain × Peasgood)]
11	Ardelean	Jonathan × Peasgood
12	Prima	The first variety resistant to *Venturia inaequalis* (Cke.) Wint. [5]
13	Roşu de Cluj	Jonathan × Senator

[1] Clar alb (the name in Romanian means in English 'Clear white'; syn. Papirovka, White Transparent, or Yellow Transparent).
[2] OP – open pollinated.
[3] Golden Pearmain O.S. – open source, abbreviation used to simplify this genotype notation, widely used in past hybridizations at HRS.

[4] N.J. – notation N.J. represent apple selections of American origin (N.J. = New Jersey), mostly complex hybrids, some of them containing the '*Vf*' gene, obtained through Romanian-American cooperation.
[5] Prima cultivar was released in 1970 from the cooperative program involving Purdue University, Rutgers University, the State University of New Jersey, and the University of Illinois designed to breed scab-resistant apples. It is heterozygous for the dominant gene *Vf* from *Malus floribunda* 821, inherited through complex hybridizations and modified backcross. Later, its monogenic resistance was defeated by new strains of the pathogen.

Table S3. Origin of apple selections or cultivars used in testcross hybridizations with the Mutsu cultivar as father, resulting in 8 hybrid combinations (offspring families).

No.	Genotype	Origin
1	218/2	(Golden Pearmain × Peasgood)]
2	X-13-10	[Aromat de vară × 218/2 (Golden Pearmain × Peasgood)]
3	X-6-73	[III-I-16-16 (Jonathan × Red Astrachan) × Raritan]
4	DSF 3/70	[III-VI-5-26 (Golden Pearmain O.S.) × N.J.46]
5	X-6-64	[III-I-16-16 (Jonathan × Red Astrachan) × Raritan]
6	X-5-71	[III-VI-5-26 (Golden Pearmain O.S.) × N.J.40]
7	Aromat de vară	See Table S1
8	Ancuţa	Jonathan × Mărul de Şugag

Table S4. Origin of apple selections or cultivars used in testcross hybridizations with the Ancuța cultivar as father, resulting in 18 hybrid combinations (offspring families).

No.	Genotype	Origin
1	X-17-16	[218/2 (Golden Pearmain × Peasgood) × Clar alb]
2	X-21-20	Reinette Baumann × *Malus niedzwetzkyana*
3	DSF 5/67	[5/3 (seeds of Jonathan O.P., irradiated) × O.P.]
4	DSF 7/68	[III-VI-5-26 (Golden Pearmain O.S.) × Camuzat]
5	DSF 3/41	[III-VI-5-26 (Golden Pearmain O.S.) × N.J.46]
6	X-6-3	[III-VI-5-26 (Golden Pearmain O.S.) × N.J.46]
7	DSF 3/80	[III-VI-5-26 (Golden Pearmain O.S.) × N.J.46]
8	DSF 5/22	[Roşu de Cluj × III-VI-20-25 (Jonathan × Senator)]
9	X-9-69	[III-VI-5-26 (Golden Pearmain O.S.) × 218/2 (Golden Pearmain × Peasgood)]
10	X-13-63	[III-I-16-16 (Jonathan × Red Astrachan) × 218/2 (Golden Pearmain × Peasgood)]
11	X-9-70	[III-VI-5-26 (Golden Pearmain O.S.) × 218/2 (Golden Pearmain × Peasgood)]
12	X-5-71	[III-VI-5-26 (Golden Pearmain O.S.) × N.J.40]
13	X-6-73	[III-I-16-16 (Jonathan × Red Astrachan) × Raritan]
14	Aromat de vară	See Table S1
15	Ardelean	Jonathan × Peasgood
16	Golden Delicious	Chance seedling, possibly a hybrid of Grimes Golden × Golden Reinette
17	Roşu de Cluj	Jonathan × Senator
18	Starkrimson	Probably a mutation of Starking Delicious

Table S5. Origin of apple selections or cultivars used in testcross hybridizations with the Prima cultivar as father, resulting in 7 hybrid combinations (offspring families).

No.	Genotype	Origin
1	X-21-20	Reinette Baumann × *Malus niedzwetzkyana*
2	X-6-24	[III-VI-5-26 (Golden Pearmain O.S.) × N.J.35]
3	X-5-65	[III-VI-5-26 (Golden Pearmain O.S.) × N.J.46]
4	X-5-52	[III-VI-5-26 (Golden Pearmain O.S.) × N.J.46]
5	X-1-20	[DSF 25/25 (Mărul de Șugag × Golden Pearmain) × Richared]
6	X-13-10	[Aromat de vară × 218/2 (Golden Pearmain × Peasgood)]
7	Ardelean	Jonathan × Peasgood

Table S6. Origin of apple selections or cultivars used in testcross hybridizations with the Starkrimson cultivar as father, resulting in 9 hybrid combinations (offspring families).

No.	Genotype	Origin
1	218/2	Golden Pearmain × Peasgood
2	III-II-17-25	Selection from hybrids of London Pepping O.P.
3	DSF 1/54	(Roşu de Cluj × Banana de iarnă)
4	DSF 5/45	Unknown origin
5	III-VI-5-26	Golden Pearmain O.S.
6	DSF 3/40	[III-VI-5-26 (Golden Pearmain O.S.) × N.J.46]
7	DSF 3/58	[III-VI-5-26 (Golden Pearmain O.S.) × N.J.46]
8	X-6-3	[III-VI-5-26 (Golden Pearmain O.S.) × N.J.46]
9	Ancuţa	Jonathan × Mărul de Șugag
